# PhenoPad: Building AI enabled note-taking interfaces for patient encounters

**DOI:** 10.1038/s41746-021-00555-9

**Published:** 2022-01-27

**Authors:** Jixuan Wang, Jingbo Yang, Haochi Zhang, Helen Lu, Marta Skreta, Mia Husić, Aryan Arbabi, Nicole Sultanum, Michael Brudno

**Affiliations:** 1 Department of Computer Science, University of Toronto, Toronto, ON Canada; 2 Vector Institute for Artificial Intelligence, Toronto, ON Canada; 3 Centre for Computational Medicine, The Hospital For Sick Children, Toronto, ON Canada; 4 DATA Team & Techna Institute, University Health Network, Toronto, ON Canada

**Keywords:** Health care, Translational research

## Abstract

Current clinical note-taking approaches cannot capture the entirety of information available from patient encounters and detract from patient-clinician interactions. By surveying healthcare providers’ current note-taking practices and attitudes toward new clinical technologies, we developed a patient-centered paradigm for clinical note-taking that makes use of hybrid tablet/keyboard devices and artificial intelligence (AI) technologies. PhenoPad is an intelligent clinical note-taking interface that captures free-form notes and standard phenotypic information via a variety of modalities, including speech and natural language processing techniques, handwriting recognition, and more. The output is unobtrusively presented on mobile devices to clinicians for real-time validation and can be automatically transformed into digital formats that would be compatible with integration into electronic health record systems. Semi-structured interviews and trials in clinical settings rendered positive feedback from both clinicians and patients, demonstrating that AI-enabled clinical note-taking under our design improves ease and breadth of information captured during clinical visits without compromising patient-clinician interactions. We open source a proof-of-concept implementation that can lay the foundation for broader clinical use cases.

## Introduction

Clinicians produce a considerable amount of data when seeing a patient, including records of patient history, physical examination, lab test requests, referral reports, etc. Some clinicians begin with free-text note-taking or structured paper forms, which are then digitized through dictation, typing into electronic health record (EHR) systems, or scanning and indexing to the patient’s records. Others prefer typing digital notes directly into EHR systems while talking with patients and polishing the drafts after the encounters.

These methods have a significant impact on clinicians’ workflow and workload. With paper-based note-taking, patient data must be registered first during the patient’s visit, and again during digitization. A large portion of such notes are unstructured and require complex transformations into computer-based documents. Indeed, clinicians spend nearly two plus hours on EHR systems per hour of direct patient care^1^. Some providers discard paper-based notes after transferring information to the EHR system, potentially losing details of their initial observations. Some hospitals maintain the original paper copies of notes, adding burden on data administration and security. With computer-based note-taking, information registration by typing is not as natural as free-hand note taking for some clinicians, decreasing efficiency and introducing errors^2^. Additionally, clinicians may pay more attention to their computers and make less eye contact with patients, creating communication barriers and reducing patient satisfaction^3–5^. Finally, the large portion of time spent on documentation outside of patient encounters is tedious and repetitive^6^, and heavy workloads can lead to dissatisfaction and burnout among clinicians, ultimately impacting their ability to provide patient care^7–11^.

Artificial intelligence (AI)-based efforts to alleviate these challenges have recently been made, including using automatic speech recognition (ASR)^12–16^, and natural language processing (NLP) technologies to transcribe patient-clinician conversations and automatically generate clinical notes^17–20^. Yet a pipeline to generate medical notes from patient-clinician conversations would require multiple ASR and NLP submodules, and would likely result in performance that is far from satisfactory.

Although modern deep learning models trained on large amounts of paired conversation transcripts and SOAP (Subjective, Objective, Assessment, and Plan) notes have stronger performance, this is usually quantitatively evaluated with standard metrics, e.g., accuracy and ROUGE (Recall-Oriented Understudy for Gisting Evaluation) scores^21^, and qualitative evaluation of usefulness and robustness in real clinical settings is lacking^22,23^. Future work is needed to alleviate existing problems of current deep learning-based approaches, such as lack of interpretability and generation of fabricated or conflicting information^23^, by creating more advanced models and large-scale annotated datasets. More importantly, robust clinical validation must be conducted before deploying these technologies into practice^24^, and the user experience of both clinicians and patients should be carefully evaluated to ensure healthcare is patient-centered^25^.

We propose a framework that integrates AI technologies actively into the interaction between patients and clinicians. We first surveyed clinicians to best understand their daily workflows, note-taking practices, views on technology in the clinic, and which note-taking system functions would be most beneficial to them. Based on the results, we developed PhenoPad, a mobile interface for free-form note-taking and standard phenotypic information capture on a tablet via a variety of modalities. Handwriting recognition (HWR), speech and speaker recognition, and clinical decision support are performed in real-time during patient-clinician conversations, and the results are sent back to clinicians immediately for validation and decision support. We then conducted a user study with physicians and patients, evaluating the practicality, efficiency, and comfort of using PhenoPad from clinician and patient perspectives. Most agreed that PhenoPad is supportive, unobtrusive, and improves patient-clinician interaction. Our findings provide guidance for future improvement of AI-based tools for clinical note-taking.

## Results

### Study Overview

**Fig. 1:**
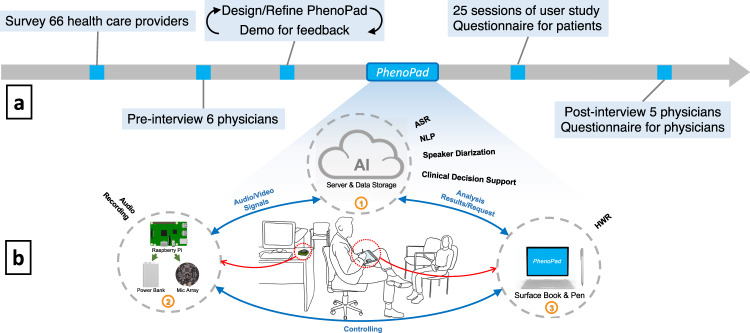
Study overview and system architecture. **a** Overview of our study methodology. **b** System architecture of PhenoPad: 1. Cloud server with data storage; 2. Audio capturing devices including a Raspberry Pi, a microphone array, and a power bank; 3. Note taking devices including a Microsoft Surface Book and a Surface Pen.

An overview of our overall study flow is illustrated Fig. 1a. We investigated the current clinical note-taking workflows and unmet needs by surveying and interviewing health care providers. Based on the results of the survey and interviews, we designed the interface and functionality of PhenoPad and iteratively refined it based on the feedback from end users. After the implementation of PhenoPad, we performed field-deployment of PhenoPad for 25 clinic visits at the Hospital for Sick Children (Toronto) and collected feedback from patients through questionnaires. Finally, we conducted semi-structured interviews with physicians who participated in our user study to further evaluate PhenoPad.

### Broad survey and interviews with clinicians

Based on the survey of 66 Ontario clinicians we found that typing directly in the EHR systems during clinical visits was the most common approach for note-taking, used by 76% of the participants. Healthcare providers’ satisfaction with their ability to capture all relevant patient information using their current note-taking practices was 3·5/5·0 and 32% of the participants reported that their current practices impair their interactions with patients sometimes to very often. This indicated room for further improvement of clinical note-taking tools and technologies.

Regarding the utility of note-taking functions, participants indicated that the ability to take photos or draw with a device were the most useful. The preference for writing notes on a tablet and recording video had the highest variance. Finally, many participants indicated that, regardless of format, it was important to seamlessly integrate the information captured into the EHR systems instead of typing manually after patient visits.

During interviews of six physicians working in the Hospital for Sick Children we identified several common problems, including: (1) making eye-contact with patients while note-taking by typing in EHR systems is difficult due to unergonomic setups in visit rooms; (2) physicians could not take notes during physical examinations since their hands are often occupied; (3) taking photos or drawing requires external devices or mediums; (4) integration of collected information into EHR systems requires extra time and effort. The full results of the survey and pre-interviews are included in Supplementary Notes 1 and 2, respectively.

### Development of PhenoPad

Based on the information we collected during the survey and pre-interviews, we designed a prototype of PhenoPad and selected several hardware devices to better support the designed functions. To design and refine the interface, we worked closely with clinicians and refined the design continuously based on clinicians’ feedback. We completed several iterations before implementing the current version of PhenoPad, shown in Fig. 1b. The source code of PhenoPad is freely and publicly available at https://github.com/data-team-uhn/PhenoPad-UWP.

Clinicians can take notes by writing on a tablet that hosts PhenoPad with a stylus. Audio capturing devices are set up in the background to collect audio signals and can be controlled remotely by PhenoPad. Data collected through various modalities, e.g., handwriting and speech, is continuously sent to a remote server for analysis, and results are sent back and presented on PhenoPad in real-time for validation and clinical feedback. All components communicate with each other wirelessly, through Wi-Fi or Bluetooth. See the “Methods” section for technical details of the implementation of the various components.

Our main considerations for selecting the hardware devices and building the infrastructures for PhenoPad were mobility, portability, versatility, and the ability to preserve privacy. After trialing multiple devices, we selected the Microsoft Surface Book with the Surface Pencil as our main note-taking devices. This was mainly due to its support of both tablet and laptop modes, allowing us to utilize it as either a tablet or as a computer. We used a Raspberry Pi powered by a portable and chargeable battery and connected with a microphone array as our audio collection device. Finally, we leveraged a server inside the hospital’s network for computationally expensive tasks and data storage.

The full workflow of using PhenoPad for clinical note-taking is as follow: during patient encounters, clinicians take note using PhenoPad in the tablet mode either from scratch or from existing history records; after patients leave, clinicians edit their notes and add more details through keyboard in the laptop mode; after being finished, the notes are sent to the EHR system. Currently, the final step is a separate step; however, in the future we hope to integrate PhenoPad directly with the EHR system. We now describe some of the key functionalities of PhenoPad.

### Efficient and comprehensive clinical information registration and capturing

PhenoPad supports multiple ways of information registration: (1) writing notes directly on the tablet using the stylus; (2) adding information that is automatically recognized from handwriting or speech; (3) drawing charts or diagrams and inserting images into notes; (4) inserting and annotating photos and videos taken with the tablet.

#### Taking notes in a natural and non-distracting way

**Fig. 2:**
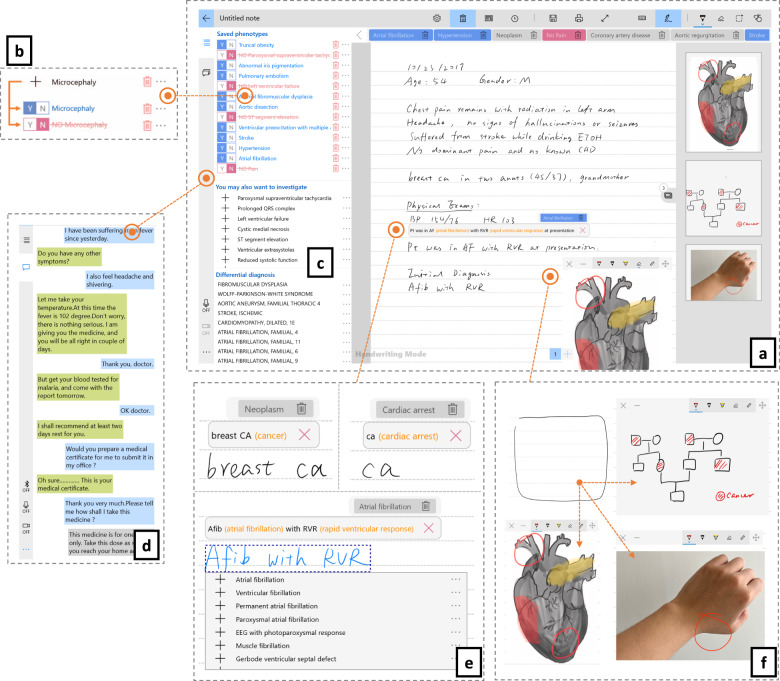
Note-taking interface design. **a** Note-taking Interface is for note-taking and information presentation. In the middle of this region is an area for taking free-form notes, written using the stylus. On the top is a menu bar for controlling and navigation. The horizontal list below this menu bar is a quick view for extracted phenotypes. **b** Phenotype Control is for phenotypic information registration. Here, clinicians can click to indicate present/absence of the corresponding phenotype. **c** Clinical Decision Support Panel and **d** Speech Transcripts Panel are two side panels that present the results of clinical decision support and speech recognition with speaker diarization. **e** Note-taking by hand is supported by HWR, abbreviation disambiguation, and phenotype extraction/searching. **f** Images, photos, and drawings can easily be inserted into the patient’s record. The user can draw a square shape for the image, photo or drawing to be added, with the desired position and size. This drawn shape will turn into different controls for sketches, images, and photos based on selection.

The PhenoPad interface for note-taking (Fig. 2a) consists of a note-taking region and a side panel for quick view of supporting information. Users can write free-structured notes directly on the tablet using the stylus. HWR, medical term extraction, and clinical abbreviation disambiguation are performed automatically in the background and the results are presented immediately to clinicians for reference and validation. HWR results show up above hand-written notes as clinicians are writing (Fig. 2e). Expanded forms of abbreviations are presented and highlighted inside these HWR results. Clicking on the words or abbreviations will pop up a list from which users can select other alternatives. Clinicians can immediately correct potential errors or make changes later. This allows clinicians to take full control of the note-taking process with as little interruption as possible.

#### Capturing and understanding patient-doctor conversations

Speech recognition and speaker diarization are performed in real-time to recognize conversation content and speakers. Audio is captured in a non-intrusive manner through a microphone array. The audio signals are then sent to the speech engine on a server, and the transcribed text and speaker diarization results are presented on PhenoPad in real time, with different colors representing different speakers (Fig. 2d). The audio is transcribed using Kaldi and we developed deep learning-based approaches to identify the speaker for every utterance^26–29^. The resulting text is fed into the NCR named entity recognition tool to identify key clinical terms^30^. This is described in more detail in the “Methods” section.

We quantitatively evaluated the ASR technology by transcribing the audio recordings collected from the user studies and used the transcribed text as ground truth for ASR evaluation. Word error rate (WER) was used as the evaluation metric, which is the ratio between the number of three kinds of errors (deletion, insertion, and substitution) and total number of words. The mean word error rate (WER) of the ASR model we used is 53·31%. We also evaluated the performance of phenotype recognition and used the phenotypes recognized from the manually transcribed text as ground truth. The precision of the phenotype recognizer we used on our collected data is 82·91% and recall is 51·08%. Detailed results are included in Supplementary Note 4.

#### Standardized phenotypic data capturing

We utilized the Human Phenotype Ontology (HPO) to standardize the phenotypic data captured from notes and speech. Other medical term collections like SNOMED or ICD-10 can also be implemented. Phenotypes are recognized and extracted from both the written notes and patient-clinician conversations automatically (Fig. 2b, e). A clinician could add a phenotype by tapping (Y) to confirm that the patient has it, or by tapping (N) to indicate that this phenotype has been investigated but not found. Clicking on the (∙∙∙) button will pop up detailed information about the phenotype, should the clinician want more information.

There is also a horizontal list of phenotypes below the menu bar serving as a quick view of extracted phenotypes from captured notes and speech. Clinicians can click on a phenotype name to add it and click it again to indicate the absence of that phenotype, should they feel that this is of importance to note. A separate interface provides a summary of the recognized phenotypes and indicates the positions where they are originally extracted.

#### Real-time clinical decision support

Real-time suggestions based on patient-clinician conversations could inform clinicians of further questions that might be useful to ask during the patient visit. PhenoPad provides *automated phenotype suggestions,* generated based on the previously selected or entered phenotypes. This suggests additional phenotypes to investigate, which can improve the patient’s phenotypic description and benefit the differential diagnosis. Similarly, *automated diagnostics assistance* suggests OMIM disorders based on the observed phenotypes. The results of these clinical decision support methods are actively updated as more information is collected during the patient visit. The choice of the ontologies here is based on an initial trial in a Genetics department, and would need to be modified for other clinics.

#### Convenient integration of drawings, images, and photos

Drawings and photos can be easily integrated into the clinician’s notes on PhenoPad through add-in panels (Fig. 2f), eliminating the need to scan hand-drawn diagrams and take photos by extra devices. Users can easily move, resize, expand space, and annotate on photos, images, or drawings. These panels can also be hidden inside a side panel (Fig. 2a).

### Starting from existing records

**Fig. 3:**
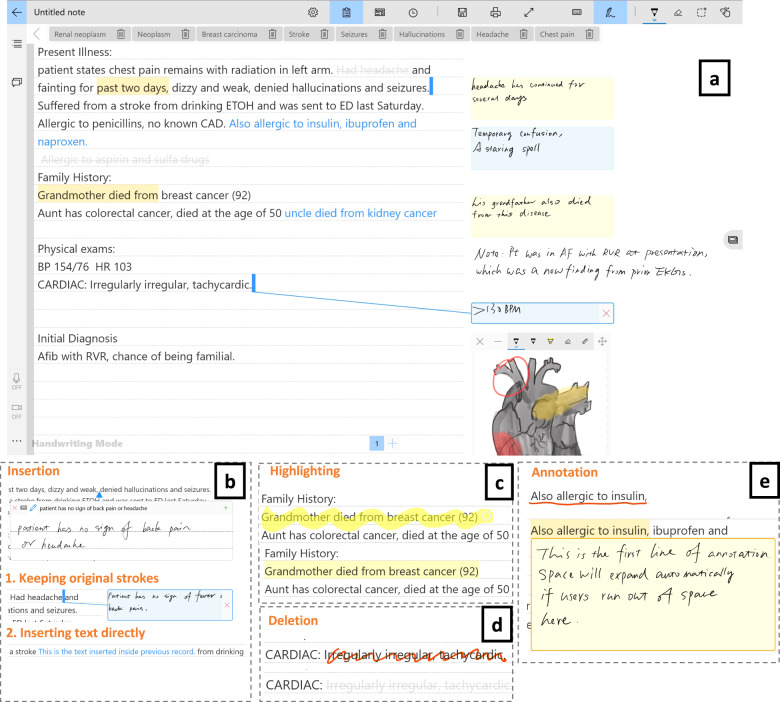
Records editing interface design. **a** Records Editing Interface is where medical history records can be imported through importing a text file or copying and pasting from another document. The panel on the right-hand side contains sticker-like controls for presenting annotated or inserted text. Hovering close to the “stickers” using the stylus without clicking them will show a line pointing back to the original positions of annotation or insertion. The blank area in the right panel can still be used for taking free-form notes. Several editing operations are supported (bottom half). **b** Insertion can be achieved by either typing or handwriting. Clinicians can: (1) keep the original hand-written strokes, which will be moved to the right-hand panel after the clinician is finished writing; (2) insert text typed or recognized from handwriting directly into history records. The inserted text will be highlighted in blue. **c** Highlighting is accomplished by pressing the side button of the stylus, such as the Surface Pencil, and “marking” the text to highlight. Releasing the side button will highlight the selected text in yellow. **d** Deletion is performed by crossing out the text to be deleted, which will then become highlighted in gray and struck through. **e** Annotation is executed by underlining the text to annotate and writing down annotations in the pop-up panel. This panel will expand automatically if more writing space is needed. Clicking anywhere outside the annotation panel will move it to the right-side panel as a golden “sticker”.

PhenoPad also allows clinicians to take note on top of already existing medical history records or those that are imported from elsewhere. This is useful for follow-up visits, or patients with records from other hospitals. Four pen gesture operations are supported for this (Fig. 3):

- Insertion: Adds new information, such as new phenotypes or medications.
- Deletion: Removes information, such as outdated or invalid records.
- Annotation: Adds comments on existing records, e.g., more details of a specific symptom, a reminder for a lab test, etc.
- Highlighting: Emphasizes parts of records with significance.

To help clinicians locate updated or important information quickly, most modifications are presented side-by-side with the medical history records. Each category of modification is presented using a unique color or marking style. Meanwhile, clinicians can still write free-form notes or insert images, photos, or diagrams in the blank area of the right side of the screen.

### Generating notes based on captured information

**Fig. 4:**
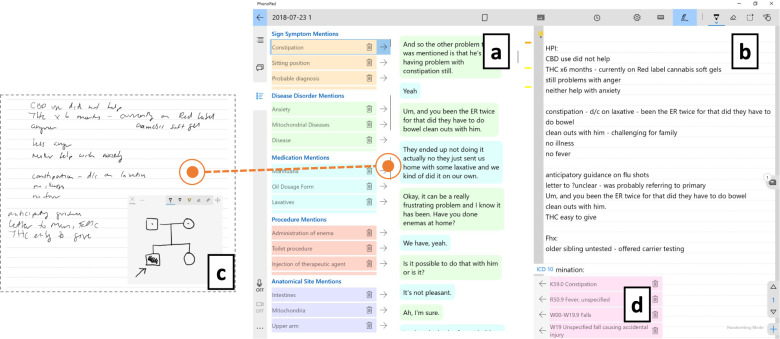
Note generation interface design. **a** Speech Transcripts Panel is for presenting the transcripts of the conversations (right) and medical information recognized from the transcripts (left). When clicking on a medical term, the positions where it appears are highlighted as yellow bars (or an orange bar if it is the one currently being shown). **b** Note Writing Panel is a regular text box for note writing and editing. Clinicians can add information from (**a**) into (**b**) by dragging and dropping the text in (**a**) into the desired position in (**b**). **c** Raw Notes Panel presents the raw notes including handwritings, drawings, photos, and/or videos. Clinicians have the option to switch between (**a**) and (**c**). **d** ICD 10 List contains a list of ICD 10 codes recognized from the conversation and used for billing purposes.

After the patient encounter, clinicians can write their notes directly on PhenoPad, using the information captured. The note generation interface presents information captured during patient encounters and the note draft side by side, as shown in Fig. 4. The note is initialized from text captured from clinician’s hand-written notes, and can be edited by typing. To reuse information from the conversations one can drag and drop the text generated by the ASR into the notes. Automatically recognized medical terms identified by the NLP are used to help the user navigate through the conversation. Automatically generated ICD-10 codes can be used to simplify clinical billing.

Currently, PhenoPad only supports partial automation of the note generation. Our end goal is to fully automate the note generation through NLP technologies, e.g., recognizing medical information, summarizing the clinical findings, generating natural language to describe the findings, grouping the generated information into different sections of the notes, etc. We leave integrating more advanced technologies for future work.

### Evaluation of PhenoPad by patients and physicians

#### Evaluation results by patients

To evaluate patient comfort and satisfaction with encounters recorded with PhenoPad we surveyed patients and caretakers immediately after clinic visits. All participants supported the use of audio recording for *specific parts* (24%) or *anytime* (76%) during their consultations. 32% of the participants indicated that they would allow video recording to be used *anytime* during consultation, 56% would allow it *only for specific parts* of the consultation and 12% requested that it *not* be used during the consultation.

**Fig. 5:**
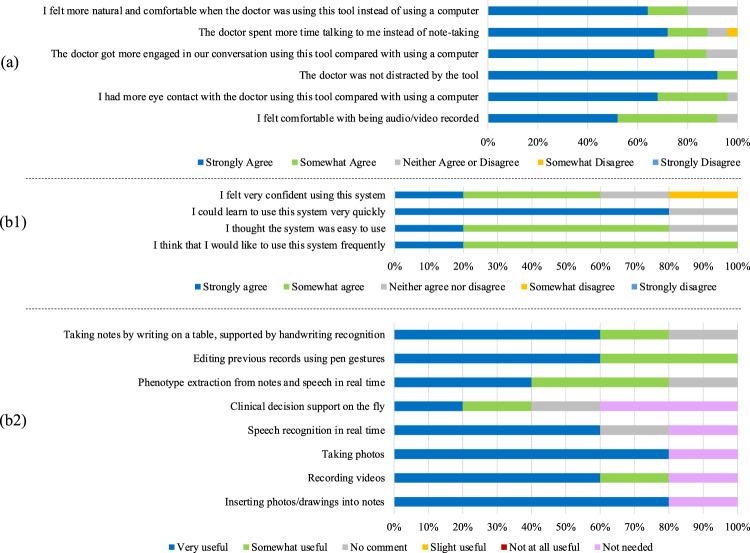
Experience of patients with PhenoPad and usability evaluation by physicians. **a** Results of the questionnaire for evaluating patients’ experience with PhenoPad. **b** Evaluation results on the usability of PhenoPad: b1 System usability scale assessment results. b2 Component-level Likert-scale assessment results.

The patients were also asked to evaluate their experience with PhenoPad during their clinic visits (Fig. 5a). The feedback we received was overall very positive, with a vast majority of participants feeling that PhenoPad allowed for more eye contact, interaction, and natural communication with physicians. Our results also demonstrated that audio/video recording was unobtrusive, as 92% of the participants indicated that they felt comfortable with the use of audio/video recording. We also received several positive comments from patients and their families (Supplementary Note 3).

#### Evaluation results by physicians

All physicians interviewed in the user study agreed that they were able to capture all necessary information using PhenoPad. They did not feel uncomfortable being surrounded by peripheral devices such as the microphone and camera. One physician stated, “*It was absolutely out of the way and was just fine for both patients and myself*.” Another suggested that the devices should be “*smaller and less visible*” since patients may be distracted by them, though this concern was not mentioned by any surveyed patients.

The physicians had generally positive attitudes and statements regarding writing notes on a tablet (Supplementary Note 3). Some however suggested additional training time to improve their ability to write on a tablet, which we recommend for future studies.

Four out of five physicians thought that audio and video recordings would be good complementary resources for clinical documentation, especially during physical examinations, as they can capture reflex exams and symptoms of movement disorders or qualitative data like emotions and facial expressions of patients. One physician summarized the benefits of this as, “*Audio means a thousand words and a video means a million words*.” Moreover, the recordings could improve communication between physicians because, as one physician explained, “*It allows other consulting physicians to see the raw data in their own eyes rather than to have to take my words for it*.”

Furthermore, we asked the physicians to compare PhenoPad with other solutions that are only based on audio, such as EmpowerMD^31^. Some users pointed out that there is still a need to write observations that are not actively verbalized in conversations or phrased differently for patients (Supplementary Note 3). On the other hand, one physician mentioned that patients may pronounce words wrongly, have rare accents, or have speech disorders, which makes their speech difficult to be recognized. Overall, the physicians agreed that it was necessary to have a note taking interface when talking to patients, in addition to any audio processing.

All physicians agreed that using PhenoPad’s note generation interface can produce notes with better quality, more details, and fewer errors. However, two of the physicians identified a trade-off between quality and speed. They suggested that typing into the computers directly, during the patient visit was the fastest way for documentation; however, this would make it more challenging to keep the focus on the patients, especially when the information content is very dense, e.g., when the patients are going through their symptoms and family history. They suggested that one potential way to accelerate the documentation speed with PhenoPad would be to populate note templates and/or routine questions automatically, based on the conversations.

More details from our semi-structured interviews can be found in the Supplementary Note 3.

We also collected quantitative evaluation results by asking the physicians to answer a short questionnaire. The results showed that physicians had generally favorable impressions and saw PhenoPad as a useful tool for clinical note-taking. When asked to rate their overall experiences from 1 (negative) to 5 (positive), the mean score from all physicians was 3·6. Using a similar 1−5 scale for the usefulness of PhenoPad, the mean score given by the physicians was 3·8.

Our questionnaire also consisted of an adapted version of the System Usability Scale (SUS) as well as a component-level Likert-scale assessment of the various features of PhenoPad to assess user perceptions in terms of usefulness, clarity, and trust (Fig. 5b). Overall, we received positive feedback that most of the PhenoPad features are useful, though some concerns were raised about some unexpected errors outputted by the system. Thus, despite some concerns with the performance of the technological components, PhenoPad showed promise as a model for future note-taking tools in the clinic.

## Discussion

In this work, we designed a clinical note-taking interface enabled by speech and NLP technologies to investigate how such a tool can help mitigate the current challenges of information capturing during patient visits. Preliminary surveys and discussions with clinicians identified issues with current workflows and guided the design of PhenoPad as an example of how AI technologies could enable more efficient and higher-accuracy clinical note-taking. PhenoPad allows clinicians to write notes in addition to ASR, instead of having to speak everything out loud as with approaches only based on speech and NLP. PhenoPad takes full advantage of speech and NLP technologies for automatic information capturing and can act as an interface for information support during patient encounters, such as presenting history records or clinical decision support. Furthermore, we open source a proof-of-concept implementation that can lay the foundation for broader clinical use cases.

Clinical documentation technology should first and foremost be unobtrusive and easily available for use when needed. Indeed, most clinicians were comfortable using PhenoPad and felt that it was straightforward and allowed them to capture the necessary information during patient visits through written notes, photo and video capture, drawing diagrams, and editing previous records, in addition to conversational recognition and clinical note generation. PhenoPad is hand-held and can be re-positioned as needed for comfort and ease of use, and its peripherals are not obtrusive. During clinic visits, patients indicated that the device did not hinder or distract them or the clinician.

Healthcare technology should be designed to put patients at the center of healthcare and allow clinicians to use the tool to steer this process during clinical visits. Through user studies in real clinical settings, we observed that PhenoPad enabled better patient-clinician interaction from both clinician and patient perspectives. Most clinicians were comfortable being audio recorded during visits, and agreed that audio recording could be useful in improving the breadth of clinical documentation. Likewise, patients largely did not object to being audio recorded for at least part of their visit, and many felt that their experience during visits was improved, as PhenoPad allowed clinicians to interact more with patients through eye contact and by focusing on the conversation as opposed to taking notes.

Nonetheless, there are several limitations to our current work. First, the capabilities and application of PhenoPad are largely limited by the technologies being used. For example, we deployed our own speech recognition system instead of using an off-the-shelf ASR model, since cloud-based ASR presented challenges regarding privacy. Additionally, most off-the-shelf solutions are for general usage and may not achieve expected performance in the clinical domain, and state-of-the-art approaches based on deep learning are data hungry. This expended a lot of engineering and research efforts, and the resulting system achieved mediocre performance by our standards. Without access to substantial amounts of in-domain datasets, which are extremely expensive to acquire, it is difficult to build or adapt models that could perform well in real clinical settings. Some technological components of PhenoPad are designed for a certain domain, e.g., the clinical decision support tools we used are for genetic diseases. These should be adopted for different types of clinics. PhenoPad leverages external APIs for different technologies and makes it easy to replace these components.

Second, certain features were favored more than others by clinicians, and adjustments should be made to better accommodate these needs. For example, integration of note-taking systems into EHR systems was considered essential by nearly all surveyed clinicians. We did not implement this in PhenoPad as it was out of scope for a research prototype. Furthermore, physicians’ opinions differed greatly on certain features depending on their personal preference, daily practice, and attitude toward new technologies. The ability to configure the tools towards specific clinical workflows is therefore critical.

## Methods

### Design methodology

We conducted a survey and pre-interviews with clinicians to better understand the current clinical workflow and clinicians’ requirement for clinical note-taking. First, we surveyed 66 health care providers with 19.5 years of practice on average, working across Ontario, Canada, on their: (1) current clinical note-taking practices; (2) views and attitude of healthcare providers towards existing and novel technologies for clinical note-taking; (3) suggestions for improving current clinical note-taking practices by new technologies. Second, we interviewed six physicians working in the Hospital for Sick Children (SickKids) in Toronto, Canada, to gain further understanding of the challenges in current note-taking workflows.

### Development of PhenoPad: System architecture

The system architecture of PhenoPad, shown in main manuscript Fig. 1b, consists of three components. Clinicians can write notes in a natural posture with the note-taking devices, which include a stylus and a tablet that hosts PhenoPad. Writing using the stylus is the main input method, although typing is also possible. We select the Microsoft Surface Book as it supports both laptop and tablet mode, and handwriting is supported by the Surface Pen. Clinicians can control the audio capturing devices—a microphone array connected to a Raspberry Pi powered by a power bank—using a Bluetooth connection via the tablet. The Raspberry Pi streams the audio signals from the microphone array to the cloud server through a secure Wi-Fi connection. The cloud server is responsible for computational tasks, including speech recognition, speaker diarization, NLP, and clinical decision support. It also serves as a cloud storage for captured and generated information. PhenoPad can send requests or receive results from the cloud server in real-time through the secure Wi-Fi connection.

### Development of PhenoPad: Implementation of data analysis pipeline

- Speech recognition: We developed and deployed a real-time speech recognition engine for medical conversations. Our ASR engine is based on Kaldi^26^. The acoustic model we used is the ASpIRE Chain Model^32^. Since there is no available language model trained specifically for medical conversations, we trained our own based on 2 million abstracts from PubMed. After cleaning up, we trimmed our vocabulary down to 82,000 unique words. The resulting ASR engine can recognize a substantial number of medical terms, including various disease names and phenotypes.
- Speaker diarization: We built our online speaker diarization system by first training a speaker embedding model on the VoxCeleb2 dataset with around 6000 speakers^33^. The embedding model, based on the Long Short-Term Memory recurrent network, is trained such that speech segments can be mapped into a fixed-dimensional embedding space in which the distance between segments from the same speakers is closer than those from different speakers^29^. Moreover, we ran beamforming algorithms on the multi-channel audio signals collected from the microphone array and combined this with speaker embeddings. This made the diarization system more robust, especially at the early stages of clinic visits when not enough enrollment data has been collected. We developed graph-based methods for speaker diarization^27,28^.
- Medical term extraction: We used Neural Concept Recognizer (NCR) for medical term extraction from notes and speech^30^.
- Abbreviation disambiguation: Our abbreviation disambiguation model was described in and trained on public medical notes^34^.
- Clinical decision support: We applied the clinical decision support methods implemented in PhenoTips^35^.

### Evaluation of PhenoPad

For evaluation, we mainly focus on a qualitative assessment of the human factors related to the use of PhenoPad, both from the provider and patient perspectives. While we also briefly evaluate some of the AI components in PhenoPad, these are quickly evolving, and the ability to use a specific component may be limited by the privacy policies of individual hospitals.

To evaluate the utility of PhenoPad we conducted 25 sessions of user study in the Emergency (17 sessions), Neurology (five sessions), Urology (two sessions), and Genetics Department (one session) of the Hospital for Sick Children with five of the six physicians whom we pre-interviewed. All sessions were audio recorded and 17 sessions were video recorded. After each session, patients and their families were given a questionnaire about their perspective on video/audio recording during their clinical visits and their evaluation of the experience with PhenoPad. The questionnaires were answered by the patients or caretakers. Of the 25 questionnaire participants, 17 were parents and eight were patients. We also conducted semi-structured interviews with the five physicians after the user studies and also asked them to complete a questionnaire to evaluate their experience with PhenoPad and the usefulness of the functionality we designed. Finally, after developing additional functionality to help clinicians document patient encounters from initial notes we conducted three additional semi-structured interviews (with physicians who had previously employed PhenoPad) to evaluate this specific component.

We also quantitatively evaluated the ASR technology by transcribing the audio recordings collected from the user studies and used the transcribed text as ground truth for ASR evaluation. To compare the performance of our ASR methodology to available commercial ASRs, we utilized a dataset of five public clinical conversations (see Supplementary Table 3), which we also manually transcribed. See Supplementary Table 4 for the comparison results.

### Ethics

Our interviews and user studies were approved by the Research Ethics Board of the Hospital for Sick Children, and our survey was approved by the Research Ethics Board of the University of Toronto. All patients and clinicians participating in the interviews and user studies provided written informed consent to take part in the study.

### Reporting summary

Further information on research design is available in the Nature Research Reporting Summary linked to this article.

## Supplementary information

Supplementary Material

Reporting Summary

## Data Availability

The survey results are included in the Supplementary Information. The audio recordings and video recordings of the clinical visits, as well as the medical notes, will not be made publicly available, and application for access to the data will require authorization of the Hospital for Sick Children Research Ethics Board (REB). Individuals employed or otherwise appointed by the Hospital for Sick Children can contact the REB at ask.crs@sickkids.ca.

## Code availability

The codes for PhenoPad are open sourced on GitHub (https://github.com/data-team-uhn/PhenoPad-UWP) and can be used by anyone to set up their own version with full functionality.
