# Supplementary material for: PhenoPad: Building AI enabled note-taking interfaces for patient encounters
Source: NPJ Digit Med. 2022 Jan 27;5:12. doi: 10.1038/s41746-021-00555-9 (PMC8795160; doi:10.1038/s41746-021-00555-9)
Supplement: Supplementary file 1 — Supplementary Material [file 41746_2021_555_MOESM1_ESM.pdf]

Supplementary Materials

Supplementary Note 1: Survey on clinical note-taking during clinic visits

We recruited two groups of healthcare providers: one group consisted of general health care practitioners (44 participants) while the other group consisted of healthcare practitioners who also serve as technical leaders (22 participants). Generally, the technical leaders are keener to and more familiar with technologies used in clinics. A majority of the participants (~68%) are physicians working in family medicine.

Current clinical note-taking practices

The primary method for clinical note-taking, reported by 76% of the participants, was typing directly into the EHR/EMR systems during clinical visits. 42% reported that they use multiple note-taking methods. 41% of the participants remember and enter into the EHR/EMR systems after the clinical visits. Only a few indicated using paper or dictation for note-taking. Moreover, some participants reported that they also use other tools, such as templates or custom forms, pre-visit questionnaires, dictation software or tools, cameras and diagram tools, to help with clinical note-taking.

Views and attitude towards current tools and novel technologies

As expected, the technical leaders' responses indicated that they are more comfortable using technologies (4·6/5·0) compared with the general practitioners (4·0/5·0). Most participants are comfortable using tablets, rated 3·9/5·0 and 4·2/5·0 by general practitioners and technical leaders, respectively. However, their ratings on comfortableness of using tablets and styluses for writing and note-taking were only 2·6/5·0 and 2·7/5·0, respectively.

Almost all the participants (95%) work with EMR or EHR systems. The satisfaction rate of the general practitioners and technical leaders of the EMR/EHR systems were 3·6/5·0 and 4·1/5·0, respectively. Moreover, their satisfaction in their ability to capture all relevant patient information using their current note-taking practices was 3·4/5·0 and 3·6/5·0, respectively and around 32% of the participants reported that their current note-taking practices impair their interactions with patients very often or sometimes. The participants' relatively low ratings of their ability to capture information using their current note-taking practices indicate room for further improvement of clinical note-taking tools and technologies.

We also asked the participants to indicate their perception of the usefulness and comfortableness of several functions we would like to include in PhenoPad. Supplemental Figure 1 shows the results of three note-taking functions. While we observed a relatively high variance of opinions on functions related to handwriting, most participants agreed that being able to edit previous notes on the tool would be useful.

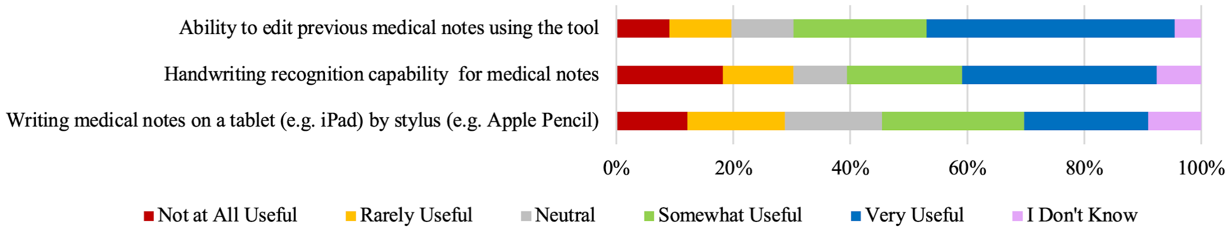

Supplemental Fig. 1 Usefulness evaluation of note-taking functions.

Supplemental Figure 2 shows the evaluation results of several audio, visual, and clinical decision support functions, most of which are considered to be very useful by the participants, with the exception of the video recording function.

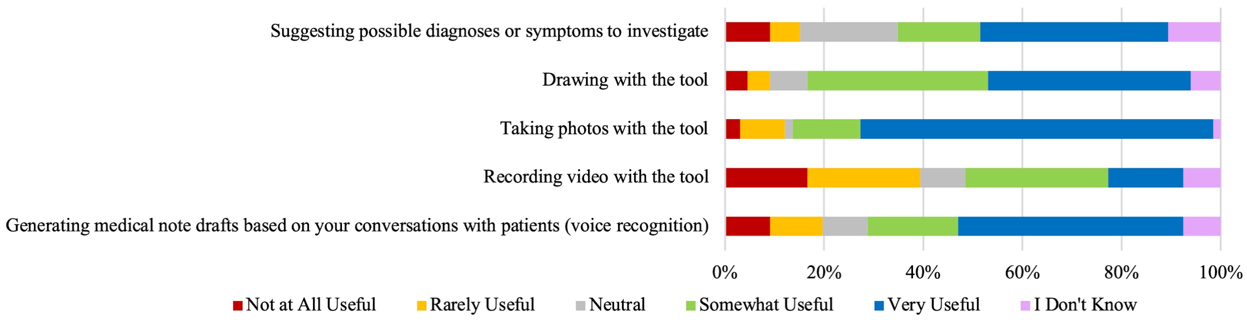

Supplemental Fig. 2 Usefulness evaluation of audio, visual, and clinical decision support functions.

We likewise asked the participants to indicate how comfortable they would be using several clinical information capturing functions shown in Supplemental Figure 3. While most functions garnered largely positive responses, writing notes on tablets, and recording video raised the most disagreement among the participants.

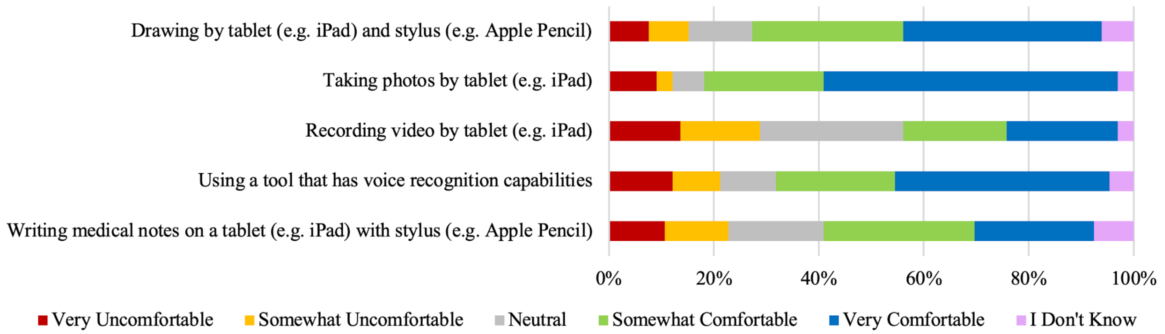

Supplemental Fig. 3 Comfortableness evaluation of using novel functions for clinical note-taking.

Suggestions for further improvement and applying novel technologies

Many participants agreed that the information captured or generated by technologies, no matter in what forms, should be seamlessly integrated into the EMR/EHR systems. They often cited that this was because typing the information into the EMR/EHR systems after clinic visits takes time, with one participant writing that they "*dread it*". Overall, the participants expressed interest in alternative solutions that could replace typing during clinic visits. As written by a participant, "*I don't type during face to face sessions since I find it noisy and harder to focus on the client, and also my desktop is at a desk and my session are across the room*", thus they think that "*to write notes on a tablet and have them automatically turn into type in the EMR would be handy.*" However, some participants said that they are less comfortable writing on tablets and a lot of participants prefer more hands-free solutions, such as technologies and tools that are able to transcribe their conversations with patients and automatically generate clinical notes. Additionally, the integrated technologies should be ergonomic, easy to use, accurate, and require low cognitive load. Besides the current functions, other preferred functions include having more customizable templates, recording and uploading audio, photos and videos, being able to draw diagrams, etc.

Supplementary Note 2: Pre-interviews with clinicians

To have a better understanding about the current practices for clinical note-taking and how technologies could fit into the current workflow, we interviewed six healthcare practitioners in the Hospital for Sick Children (one from the Genetics Department, four from the Neurology Department, and one from the Emergency Department). All interviews were audio recorded. We summarize the interview results below.

A typical patient visit consists of three stages: preparation before the patient arrives, the clinical visit itself, and after the patient has left. We found several problems in this workflow that either increase the workload of clinicians or disturb the interaction between patients and clinicians. Moreover, there were several requirements that could not be perfectly satisfied by using the current tools available in hospitals.

Preparation for a patient’s clinic visit

For some departments, clinicians need to prepare for patient visits beforehand by reviewing patients’ histories and their health records. Some clinicians rely entirely on their memory, while others prepare an information sheet to refer to when talking with their patients. There were several challenges that the clinicians we interviewed brought up regarding these methods. For example, medical records such as images, lab tests, and clinical notes, are often stored across various systems that clinicians must use to get the whole picture of their patients’ histories and medical records. Regarding this, some clinicians said, “*it's hard to navigate between different systems.*” They also spoke about their challenges with using preparation sheets printed on paper. “*I print the preparation sheet. And then I write on the paper with the comments on top of what I have prepared before.*” These sheets include limited space for writing and require extra efforts to transform the written notes back into a digital format. Furthermore, sometimes multiple health care providers will work together to create the preparation sheet for efficiency. “*One person looks for information and another person writes it down,*” explained the clinicians. This, however, requires efficient communication and collaboration.

During a patient’s clinic visit

During clinic visits, clinicians discuss patients’ their health conditions and concerns and conduct physical examinations. Topics being discussed include history of present illness, allergy, family history, assessment, care plan, and so on. Some clinicians memorize everything without taking any notes, but most clinicians depend heavily on note-taking. “*If I don’t take notes, I will forget everything after four or five patients,*” said one clinician that we spoke with.

In current practice, clinicians usually type the notes directly into the EHR system. However, we observed that the current setup in many clinic visit rooms are not ergonomically well-designed and could lead to unnatural physical position for typing. Clinicians usually sit between monitors and patients, and regarding this, some clinicians said, “*It’s tricky to make eye contact with the patients while typing.*” Furthermore, during physical examination, it is difficult for clinicians to take any notes since both of their hands are often occupied, and they usually stand away from the computers. This means that they often must remember everything from the patient encounter until they are able to make note of it either during the visit, taking time away from the patient, or afterwards at the risk of forgetting critical details.

Others rely on paper-based note-taking, using either blank paper or pre-made preparation sheets. To make hand-writing easier and faster, many clinicians use abbreviations, for example, “no/H” to indicate “no headache”. As there is no standard set of medical abbreviations, different clinicians may use different short-forms for the same term. This, in addition to difficulty in recognizing others’ handwriting, can make sharing of notes between various clinicians more difficult, impinging on integrated patient care. Furthermore, it is hard to refer to history records that are on loose-leaf paper. Some clinicians mitigate this by using the pre-printed preparation sheets. They would “*pull arrows from a part of the prepared text and add notes there*”, which might cause poor organization of medical notes and make patient record-keeping difficult.

Clinicians also have multimedia requirements that cannot be easily met by current set-ups, which was also indicated by many clinicians in the survey. For example, they must “*grasp a camera*” to take photos, which have to be imported into the EHR system afterwards. They also often “*show patients images, like MRI images,*” on monitors, which leads to uncomfortable physical positions for both the clinician and the patient viewing the images. Some clinicians “*draw a lot*” for education, illustration or documentation purposes, which requires additional separate papers and is inconvenient to integrate into digitized clinical notes.

Following a patient’s clinic visit

After patients have left the clinic, clinicians usually must spend time elaborating their memory or the notes they have taken into medical stories and type them into the EHR system. High volumes of information that must be remembered and noted or transcribed for various patients present challenges to clinicians’ memory and require extra time and efforts to record, often outside of standard work hours.

Supplementary Note 3: Evaluation of PhenoPad

Feedbacks from patients

We also received several positive comments from patients and their families. For instance, one patient wrote "*this tool will be very beneficial to doctors to use*", another patient wrote: "*did not notice/worry about the audio/video tool. It was not intrusive*", and two parents wrote that it was a "*great experience*".

Feedbacks from physicians

On note-taking on tablets

One physician said, "*It fits me well like what paper did; it works very similarly with papers*," while another said, "*My physical positioning and ability to engage with the patients was improved from being anchored to the computer.*" However, some physicians mentioned that they would appreciate additional training time to improve their ability to write on a tablet: "*I'm more efficient with writing on papers and I definitely need practice for writing on the tablet.*" In future studies we recommend a longer training period to get clinicians comfortable with tablets.

On audio/video functions

One physician summarized the benefits of audio and video recordings as, "*Audio means a thousand words and a video means a million words.*" Another physician said that video recording would enable him to work together with other physicians afterwards and is a richer way to make better decisions.

All physicians believed the audio recording and speech recognition features are useful for the documentation of physical examinations. Because they have to leave the computers and any note-taking tools during that time. They usually remember the details by memory and sometimes take quick notes on their hands. The speech capturing and recognition ability of PhenoPad can be helpful in this case. One physician mentioned that they would like to speak out about what they are doing and their findings to keep the patients and families engaged, and what they say can be transcribed and populated into their notes. However, another physician raised privacy-related concerns about speaking everything out.

On tools only based on audio

One physician gave an important example of why only audio is not enough, saying, "*sometimes you may want to write things down that you're not actively verbalizing in the conversations.*" Another physician also stated, "*I will verbalize differently talking to patients with what I write in my notes*."

On the note generation interface

All of them think that writing notes using this interface can produce notes with better quality, in the sense that it provides much more details and potentially decreases errors: "what is useful is that you can go back and identify things you missed." Although most prefer that the content of the conversations can be automatically populated into the notes, they agree that being able to grasp information from the conversation and put it into the notes manually is a useful feature. Moreover, being able to record videos and play them when writing notes is very helpful for use cases like recording movement disorders.

Two of the physicians point out that there might be a trade-off between quality and speed. With PhenoPad, there are more details to review and extra steps before sending the notes into the EMR systems. They have been used to typing into the EMR systems when talking to the patients and families, and one of them could type very fast even without looking at the keyboard. Typing directly into the computers might be the fastest way for documentation but, as mentioned by one physician, it will prevent physicians from keeping their focus on the patients, especially when the information to record is very tense, e.g., when the patients are going through their symptoms and family history.

One suggestion that could potentially accelerate the speed greatly is populating notes templates and/or routine questions automatically based on the conversations. When they are discussing with the patients, the interface can automatically summarize the discussion and put it into the right section, and fill in the routine questions that need to be asked for every patient in the clinic, like whether the patient has a headache or how many hours the patient sleeps everyday.

Other suggestions they gave include transforming spoken language into written language by generating paragraphs instead of just concepts, suggesting further questions to ask, sharing the conversation transcripts with patients for reference purposes, etc.

Supplementary Note 4: Evaluation results of the applied technologies

In this section, we report the evaluation results of some technologies we used to build PhenoPad.

ASR evaluation results

We evaluated the ASR performance of each session with manually transcribed text as the ground truth. Evaluation results are shown in Supplementary Table 1. The overall WER across all sessions is 53·31%. The main reason for such a high error rate is lack of in-domain data for model training or adaptation. As a result, our ASR system suffers from background noises, overlapping speech, rapid speaker change and speaker accents.

Session ID	Total Errors #	Total Words #	WER/%
1	743	2254	32·96%
2	2041	3377	60·44%
3	1940	3286	59·04%
4	1466	2390	61·34%
5	1359	2308	58·88%
6	1316	2507	52·49%
7	1201	2327	51·61%
8	573	1101	52·04%
9	630	952	66·18%
10	197	347	56·77%
11	1684	3182	52·92%
12	1581	3178	49·75%
13	717	1281	55·97%
14	875	1627	53·78%
15	794	1723	46·08%
16	590	1069	55·19%
17	923	1674	55·14%
18	1135	2933	38·70%
19	298	396	75·25%
20	547	1009	54·21%
21	580	1125	51·56%
22	1015	1801	56·36%
23	953	1631	58·43%
24	1198	1997	59·99%
25	467	1092	42·77%
**Overall**	**24823**	**46567**	**53·31%**

Supplementary Table 1. ASR evaluation results. # refers to count number.

Medical term recognition performance

Following are the evaluation results of medical term recognition. We used the medical terms recognized from the manually transcribed text as ground truth, and those recognized from the ASR outputs as predictions· In average, the mean precision for medical term recognition is 82·91% while the mean recall is 51·08%.

Session	True Positive #	False Positive #	Prediction #	GT #	Precision	Recall
1	9	1	10	12	90·00%	75·00%
2	6	5	11	19	54·55%	31·58%
3	8	1	9	27	88·89%	29·63%
4	9	1	10	15	90·00%	60·00%
5	6	3	9	12	66·67%	50·00%
6	2	2	4	11	50·00%	18·18%
7	3	3	6	16	50·00%	18·75%
8	2	0	2	6	100·00%	33·33%
9	1	2	3	7	33·33%	14·29%
10	0	0	0	2	N/A	0·00%
11	13	1	14	16	92·86%	81·25%
12	7	0	7	15	100·00%	46·67%
13	6	0	6	8	100·00%	75·00%
14	9	1	10	21	90·00%	42·86%
15	11	4	15	14	73·33%	78·57%
16	1	2	3	4	33·33%	25·00%
17	11	1	12	19	91·67%	57·89%
18	16	0	16	18	100·00%	88·89%
19	2	0	2	6	100·00%	33·33%
20	10	0	10	13	100·00%	76·92%
21	10	0	10	15	100·00%	66·67%
22	6	2	8	14	75·00%	42·86%
23	7	0	7	13	100·00%	53·85%
24	6	2	8	10	75·00%	60·00%
25	4	3	7	10	57·14%	40·00%
**Summary**	**165**	**34**	**199**	**323**	**82·91%**	**51·08%**

Supplementary Table 2. Medical term recognition evaluation results. # refers to the count number. GT refers to ground truth.

Public medical conversations

Session	Title	URL
1	History Taking 1	https://youtu.be/vV1CIF1zK0c
2	History Taking 2	https://youtu.be/GZAUlT2ujcw
3	Examination of the eyes	https://www.youtube.com/watch?v=0OzB_bqOAps
4	Charcot-Marie-Tooth (CMT) - Neurological Examination, Presentation and Parental Interview	https://www.youtube.com/watch?v=Wb7VTTNMhyM&t=179s
5	History-taking and physical examination couplet station	https://www.youtube.com/watch?v=EbdMiNRaZJg

Supplementary Table 3. Public clinical conversations we used for comparing our ASR model with commercial ASR models.

ASR performance on public medical conversations

Session	Duration	PhenoPad (Kaldi, real-time)			Google speech API (real-time)			Youtube Subtitle (offline)		
		Total Errors	Total Words	WER %	Total Errors	Total Words	WER %	Total Errors	Total Words	WER %
1	0:18:01	1659	3340	49.67%	1720	3258	52.79%	426	3305	12.89%
2	0:09:31	655	1497	43.75%	523	1437	36.40%	125	1460	8.56%
3	0:06:41	580	982	59.06%	489	975	50.15%	214	979	21.86%
4	0:14:40	1197	1874	63.87%	1039	1853	56.07%	442	1869	23.65%
5	0:07:27	333	1254	26.56%	404	1232	32.79%	92	1245	7.39%
**Sum**	0:56:20	4424	8947	49.45%	4175	8755	47.69%	1299	8858	14.66%

Supplementary Table 4. ASR evaluation results on publicly available patient encounter recordings. We compared the model we use in PhenoPad (Kaldi, streaming) with Google speech API (streaming) and the subtitles generated by Youtube, which could be seen as the off-line results of Google's ASR pipeline.
