# Supplementary material for: PhenoPad: Building AI enabled note-taking interfaces for patient encounters
Source: NPJ Digit Med. 2022 Jan 27;5:12. doi: 10.1038/s41746-021-00555-9 (PMC8795160; doi:10.1038/s41746-021-00555-9)
Supplement: Supplementary file 2 — Reporting Summary [file 41746_2021_555_MOESM2_ESM.pdf]

nature portfolio

Corresponding author(s): Jixuan Wang, Michael Brudno

Last updated by author(s): Dec 10, 2021

Reporting Summary

Nature Portfolio wishes to improve the reproducibility of the work that we publish. This form provides structure for consistency and transparency in reporting. For further information on Nature Portfolio policies, see our Editorial Policies and the Editorial Policy Checklist.

Statistics

For all statistical analyses, confirm that the following items are present in the figure legend, table legend, main text, or Methods section.

n/a	Confirmed	
☒	☐	The exact sample size (*n*) for each experimental group/condition, given as a discrete number and unit of measurement
☒	☐	A statement on whether measurements were taken from distinct samples or whether the same sample was measured repeatedly
☒	☐	The statistical test(s) used AND whether they are one- or two-sided<br>*Only common tests should be described solely by name; describe more complex techniques in the Methods section.*
☒	☐	A description of all covariates tested
☒	☐	A description of any assumptions or corrections, such as tests of normality and adjustment for multiple comparisons
☒	☐	A full description of the statistical parameters including central tendency (e.g. means) or other basic estimates (e.g. regression coefficient) AND variation (e.g. standard deviation) or associated estimates of uncertainty (e.g. confidence intervals)
☒	☐	For null hypothesis testing, the test statistic (e.g. *F*, *t*, *r*) with confidence intervals, effect sizes, degrees of freedom and *P* value noted<br>*Give P values as exact values whenever suitable.*
☒	☐	For Bayesian analysis, information on the choice of priors and Markov chain Monte Carlo settings
☒	☐	For hierarchical and complex designs, identification of the appropriate level for tests and full reporting of outcomes
☒	☐	Estimates of effect sizes (e.g. Cohen's *d*, Pearson's *r*), indicating how they were calculated

Our web collection on statistics for biologists contains articles on many of the points above.

Software and code

Policy information about availability of computer code

Data collection	The source code of our project is freely and publicly available at https://github.com/data-team-uhn/PhenoPad-UWP
Data analysis	The codes we used for data analysis are also available at the above Github repository.

For manuscripts utilizing custom algorithms or software that are central to the research but not yet described in published literature, software must be made available to editors and reviewers. We strongly encourage code deposition in a community repository (e.g. GitHub). See the Nature Portfolio guidelines for submitting code & software for further information.

Data

Policy information about availability of data

All manuscripts must include a data availability statement. This statement should provide the following information, where applicable:

- Accession codes, unique identifiers, or web links for publicly available datasets
- A description of any restrictions on data availability
- For clinical datasets or third party data, please ensure that the statement adheres to our policy

The data we collected, including audio recordings, video recordings, and medical notes, will not be made publicly available. The codes for PhenoPad are open sourced on GitHub (https://github.com/data-team-uhn/PhenoPad-UWP) and can be used by anyone to set up their own version with full functionality.

Field-specific reporting

Please select the one below that is the best fit for your research. If you are not sure, read the appropriate sections before making your selection.

☐ Life sciences ☒ Behavioural & social sciences ☐ Ecological, evolutionary & environmental sciences

For a reference copy of the document with all sections, see nature.com/documents/nr-reporting-summary-flat.pdf

Behavioural & social sciences study design

All studies must disclose on these points even when the disclosure is negative.

Study description	Our study mainly consists of surveys, user studies, questionnaires and interviews.<br>We did a survey with health care providers, user studies with patients and clinicians, and questionnaire/interviews with the user study participants, from which we collected both qualitative and quantitative data.
Research sample	We surveyed 66 health care providers with 19.5 years of practice on average, working across Ontario. □□<br>We interviewed six physicians working in the Hospital for Sick Children (SickKids) in Toronto, Canada.<br>We conducted 25 sessions of user study in the Emergency (17 sessions), Neurology (5 sessions), Urology (2 sessions), and Genetics Department (1 session) of the Hospital for Sick Children with five of the six physicians whom we pre-interviewed.<br>All participants were randomly chosen and decided whether to participate on their own will.
Sampling strategy	The participating clinicians were self-selected based on their interest in the research area. Patients approached were ones seen on specific clinic days and not pre-selected.
Data collection	For in-person study, clinicians' handwriting notes were collected by our developed interface. The conversations between patients and clinicians were recorded by microphones and cameras that were set up in the room.<br>No one else was present besides the participants (patient, provider) and the researcher.<br>The researcher was blind to the study hypothesis during data collection.
Timing	Data was collected in 2019 and 2020.<br>Several more user studies were conducted in 2021, after receiving request for major revision.
Data exclusions	No data was excluded.
Non-participation	We did not count the number of participants who declined to participate. No participant withdrew after agreeing to participate.
Randomization	No experimental group was formed.

Reporting for specific materials, systems and methods

We require information from authors about some types of materials, experimental systems and methods used in many studies. Here, indicate whether each material, system or method listed is relevant to your study. If you are not sure if a list item applies to your research, read the appropriate section before selecting a response.

Materials & experimental systems

n/a	Involved in the study	
☒	☐	Antibodies
☒	☐	Eukaryotic cell lines
☒	☐	Palaeontology and archaeology
☒	☐	Animals and other organisms
☐	☒	Human research participants
☒	☐	Clinical data
☒	☐	Dual use research of concern

Methods

n/a	Involved in the study	
☒	☐	ChIP-seq
☒	☐	Flow cytometry
☒	☐	MRI-based neuroimaging

Human research participants

Policy information about studies involving human research participants

Population characteristics	See above.
Recruitment	The participating physicians self-selected based on interest. Patients were selected based on being seen on specific clinic days, without any pre-selection.

Ethics oversight	Hospital for Sick Children and the University of Toronto

Note that full information on the approval of the study protocol must also be provided in the manuscript.
